# ﻿Interesting mycoparasites and *Paradingleyomyceslepidopterorum* gen. et sp. nov. (Hypocreales, Polycephalomycetaceae) from Yunnan Province, China

**DOI:** 10.3897/mycokeys.110.134132

**Published:** 2024-11-15

**Authors:** Yi Wang, De-Ping Wei, Xing-Can Peng, Ji-Chuan Kang, Zeng-Zhi Li, Chun-Ru Li, Xian Zhang, Gui-Ying Wang, Yun Zhou, Xin-Sheng He, Putarak Chomnunti, Ting-Chi Wen

**Affiliations:** 1 School of Pharmacy, Guizhou University, Guiyang 550025, Guizhou, China; 2 State Key Laboratory of Green Pesticide, Key Laboratory of Green Pesticide and Agricultural Bioengineering, Ministry of Education, Guizhou University, Guiyang 550025, China; 3 Engineering Research Center of Southwest Bio-Pharmaceutical Resources, Ministry of Education, Guizhou University, Guiyang 550025, China; 4 Center of Excellence in Fungal Research, Mae Fah Luang University, Chiang Rai 57100, Thailand; 5 School of Science, Mae Fah Luang University, Chiang Rai 57100, Thailand; 6 Zhejiang BioAsia Life Science Institute, Pinghu 314200, China; 7 School of Life Science and Engineering, Southwest University of Science and Technology, Mianyang 621010, China

**Keywords:** Entomopathogenic fungi, new genus, phylogeny, taxonomy

## Abstract

A novel genus, *Paradingleyomyces* was introduced to accommodate *Pa.lepidopterorum***sp. nov.**, based on a multigene phylogenetic analysis and its distinct morphological characteristics. Maximum likelihood (ML) and Bayesian inference analyses (BI) of ITS, SSU, LSU, *tef-1α*, *rpb1*, and *rpb2* sequence data shown that *Pa.lepidopterorum* formed an independent lineage nested between *Perennicordyceps* and *Dingleyomyces*. Morphologically, *Paradingleyomyces* is distinguished from *Perennicordyceps* by the presence of a white subiculum on the stromata of Ophiocordycepscf.cochlidiicola. Perithecia are produced sporadically from the base to the apex of the stromata, and the secondary ascospores exhibit a notable length-to-width ratio. These characteristics distinguish *Paradingleyomyces* from *Perennicordyceps* which exhibits tortuous, branched, clavate to cylindrical stromata with rhizomorphs, parasitism of coleopteran and hemipteran larvae, and colonizes a broader range of fungal hosts. Additionally, perithecia in *Perennicordyceps* typically arise from the middle to the upper regions of the stromata, with secondary ascospores displaying a comparatively lower length-to-width ratio. *Paradingleyomyces* is morphologically identical to *Dingleyomyces* in its direct production of superficial perithecia on the stromata of *Ophiocordyceps* species. However, the phylogenetic analysis indicates that *Paradingleyomyces* and *Dingleyomyces* are not congeneric. Moreover, this study introduces another novel species, *Polycephalomycestengchongensis*, and a novel sexual morph of *Pleurocordycepsyunnanensis*. Dimorphic phialides and conidia of *Pleurocordycepsparvicapitata* were observed and described for the first time based on a fresh collection from Tengchong County, Yunnan Province, China.

## ﻿Introduction

*Polycephalomyces* was introduced as an entomopathogenic genus by Kobayasi (1941), based on the asexual morph of the *Po.formosus*, which was characterized by polycephalous synnemata with white to pale yellow conidial masses on the tips. Species of *Polycephalomyces* form parasitic associations with a wide range of hosts, including insects, fungi and myxomycetes (Shrestha et al. 2017; Xiao et al. 2023). Among these, *Ophiocordyceps* species are the most common hosts to *Polycephalomyces*. For instance, *Polycephalomycessinensis* was found as a hyperparasite on *Ophiocordycepssinensis*, *Po.ramosus* on *Hirsutellaguignardii* and *Pleurocordycepslianzhouensis* on *O.crinalis* (Massee 1895; Kobayasi 1941; Kepler et al. 2013; Wang et al. 2014). Members of *Polycephalomyces* were expanded to include sexual species that were phylogenetically distant from the *Ophiocordyceps* sensu stricto by Kepler et al. (2013). In this study, the sexual morph of *Polycephalomyces* was described as possessing ﬁrm, pliant, multifurcating stromata, perithecia that are either superficial or immersed in an apical or subapical pulvinate cushion, filiform asci and disarticulating ascospores. The taxonomic placement of *Polycephalomyces* remained uncertain until Quandt et al. (2014) classified it within the family Ophiocordycipitaceae based on phylogenetic analyses of combining SSU, LSU, *tef-1α*, *rpb1* and *rpb2* sequence. *Perennicordyceps* was later established by Matočec et al. (2014) to accommodate four species (*Perennicordycepscuboidea*, *Pe.paracuboidea*, *Pe.prolifica*, and *Pe.ryogamiensis*), which were previously classified under *Polycephalomyces*, based on comprehensive morphological characteristics and molecular data analyses. *Perennicordyceps* is characterized by the presence of superficial perithecia and acremonium-like or hirsutella-like asexual morphs (Matočec et al. 2014; Hyde et al. 2018; Wei et al. 2022). Wang et al. (2021) established *Pleurocordyceps* to accommodate ten species that previously were placed in *Polycephalomyces*. An increasing number of polycephalomyces-like species have been added to *Polycephalomyces*, *Perennicordyceps*, and *Pleurocordyceps*, contributing to a more refined understanding of their natural classification and phylogenetic relationships (Wang et al. 2014; Wang et al. 2015a, 2015b; Liang et al. 2016; Crous et al. 2017a; Xiao et al. 2018, 2023; Yang et al. 2020). Xiao et al. (2023) constructed a backbone tree of Hypocreales using extensive taxon sampling, and the result clearly showed that *Polycephalomyces*, *Perennicordyceps*, and *Pleurocordyceps* form a monophyletic clade distinct from Ophiocordycipitaceae. As a result, a new family, Polycephalomycetaceae, was established to accommodate these three genera. It is worth noting that in the phylogenetic analysis conducted by Xiao et al. (2023), Polycephalomycetaceae was identified as a sister clade with Ophiocordycipitaceae. However, in the study conducted by Wei et al. (2022), Polycephalomycetaceae was found to form a sister clade to Clavicipitaceae. These contrasting findings suggest that the phylogenetic relationship between Polycephalomycetaceae and other hypocrealean families requires further confirmation through future research, incorporating more comprehensive taxon sampling. Therefore, discovering hidden or yet unknown species within Polycephalomycetaceae is essential for improving our understanding of this family’s evolutionary position.

During our ongoing exploration of the diversity of entomopathogenic fungi and their associated fungi in Yunnan Province, China, several polycephalomyces-like species were found from various hosts including *Elaphomyces* sp., lepidopteran larvae, *Ophiocordycepsnutans*, and Perennicordycepscf.elaphomyceticola. This study aims to assess the phylogenetic relationships of these samples with existing species of Polycephalomycetaceae using a concatenated SSU, ITS, LSU, *tef-1α*, *rpb1* and *rpb2* sequences, as well as detailed morphological analyses. The morphological observations and phylogenetic analyses allowed us to introduce a new genus, *Paradingleyomyces*, a new species, *Polycephalomycestengchongensis*, a new sexual morph of *Pleurocordycepsyunnanensis*, and a new collection of *Pleurocordycepsparvicapitata*. These findings expand our understanding of this unique group of entomopathogens and mycoparasites, offering fresh and novel insights into their morphology, ecology and evolutionary relationships.

## ﻿Materials and methods

### ﻿Morphological study and isolation

To explore the diversity of fungal resources, samples were collected from tropical and subtropical forests rich in evergreen trees diversity in southwestern China. Morphological studies followed the guidelines proposed by Senanayake et al. (2020). Specimens were placed in small plastic boxes and transported to the laboratory for isolation. Both specimens and colonies were photographed using a Canon 6D camera equipped with a 100 mm MACRO lens for detailed morphological documentation. Fruiting bodies were examined, and free-hand sections were prepared using a stereomicroscope (Leica S9E). Slides containing sectioning of the fertile parts were mounted for microscopic observation with a Leica DM2500 compound microscope equipped with a digital camera. Micro-morphological characters, including ascomata, perithecia, peridium texture, asci, ascospores, secondary ascospores, conidiophores, phialides, and conidia were photographed and measured by using the Leica microsystem for precise documentation and analysis. A small mass of tissue from the fertile parts of the fungus or insect bodies was transferred to potato glucose agar (PDA) plate using sterile inoculation needles and incubated at 25 °C to obtain pure isolates (Wen et al. 2014; Aini et al. 2020; Peng et al. 2024). Freshly collected specimens were dried using silica gel to preserve them as dry specimens. The cultures were deposited in the Herbarium of Kunming Institute of Botany Culture Collection (KUNCC; http://english.kib.cas.cn/) and the dry specimens were deposited in the Herbarium of Cryptogams, Kunming Institute of Botany of the Chinese Academy of Sciences (KUN; http://kun.kingdonia.org/).

### ﻿DNA extraction, PCR amplification and sequencing

DNA was extracted from fresh specimens and cultures using the E.Z.N.A.^TM^ Fungal DNA MiniKit (Omega Biotech, CA, USA) following the manufacturer’s protocols. Polymerase chain reaction (PCR) was performed to amplify six loci: the small subunits nuclear of rDNA (SSU), the internal transcribed spacer (ITS), the large subunit nuclear of rDNA (LSU), the transcription elongation factor-1α (*tef-1α*), the partial RNA polymerase II largest subunit (rpb1) and the partial RNA polymerase II second largest subunit (*rpb2*). The primer pairs used for amplifying the six loci were as follows: NS1 and NS4 for SSU (White et al. 1990), ITS5 and ITS4 for ITS (White et al. 1990), LROR and LR5 for LSU (Vilgalys and Hester 1990), 983F and 2218R for *tef-1α* (Rehner and Buckley 2005), CRPB1A and RPB1Cr for *rpb*1 (Castlebury et al. 2004), and RPB2-5F and RPB2-7R for *rpb2* (Liu et al. 1999). Amplification reaction was performed in a 50 μL reaction volume containing 4 μL of DNA template, 2 μL of each forward and reverse primers (10 pM), 22 μL of 2× Taq PCR StarMix with Loading Dye (GenStar) and 20 μL of twice-sterilized water. The amplification conditions for ITS, LSU, SSU, *tef-1α*, *rpb1*, and *rpb2* were as follows: (1) 3 min at 94 °C, (2) 33 cycles of denaturation at 94 °C for 30 s, annealing (ITS at 52 °C for 50 s, SSU at 47 °C for 1 min 20 s, LSU at 50 °C for 30 s, *tef-1α* at 58 °C for 50 s, *rpb1* and *rpb2* at 51 °C for 40 s), and elongation (ITS at 72 °C for 45 s, SSU and LSU at 72 °C for 1min 50 s, *tef-1α* at 72 °C for 1 min, *rpb1* and *rpb2* at 72 °C for 1 min 20 s), and (3) final extension at 72 °C for 10 min. The PCR products were sent to Tsingke Biological Technology in Chongqing, China, for sequencing, and the resulting sequences were submitted to GenBank for the assignment of accession numbers.

### ﻿Phylogenetic analysis

The newly generated sequences were checked and assembled using BioEdit v.7.0.5.3 (Hall et al. 2011). The assembled sequences were then subjected to BLAST searches in the GenBank database of National Center for Biotechnology Information (NCBI) to confirm their identities. Taxa used for phylogenetic analyses were chosen based on relevant publications and presented in Table 1. The individual gene was aligned using MAFFT (Katoh and Standley 2013). Trimal v1.2 was used to remove alignments sites that did not achieve a user specified gap score of 0.6 (Capella-Gutiérrez et al. 2009). The trimmed alignments were concatenated using FasParser 2.10.0 (Sun 2017). The final combined alignment was subjected to Maximum likelihood (ML) and Bayesian inference (BI) analyses. ML analysis was performed using IQ-TREE 1.6.12, with branch support estimated from 1000 ultrafast bootstraps replicates (Minh et al. 2020).MrModelTest v. 2.3 (Nylander 2004) was used to determine the best evolutionary model for Bayesian inference analysis according to the Akaike Information Standard (AIC). The best-fit models GTR+I+G, were determined for SSU, ITS, LSU, *tef1-α, rpb1*, and *rpb2*. The BI analysis was carried out using MrBayes on XSEDE (3.2.7a) through the CIPRES Science Gateway V 3.3 platform (Miller et al. 2010). Four Markov chain Monte Carlo (MCMC) simulations were run for 50,000,000 generations, sampling every 1000 generations and discarding the first 25% as burn-in. The remaining trees were used to calculate Bayesian posterior probabilities. The resulting trees were visualized using FigTree v1.4.3 (Rambaut 2012). To determine whether the taxa represented new species or new records, the guidelines of Jeewon and Hyde (2016) were followed.

**Table 1.** Accession numbers of taxa used in this study. Newly generated sequences are indicated in bold. ^T^ Represents type strain, type specimens or neotype.

Current name	Voucher	SSU	ITS	LSU	*tef-1α*	*rpb1*	*rpb2*	Reference
*Dingleyomyceslloydii*	PDD1212154^T^	OR647563	OR602634	OR602640	OR588853	OR588860	OR588858	Johnston and Park (2023)
***Paradingleyomyceslepidopterorum***	**HKAS 131926** ^T^	–	**OR878363**	**OR828238**	–	**OR829674**	**OR880683**	**This study**
***Paradingleyomyceslepidopterorum***	**HKAS 131927**	–	**OR878364**	**OR828239**	**OR880679**	**OR829675**	–	**This study**
***Paradingleyomyceslepidopterorum***	**HKAS 131921**	–	–	**OR828242**	–	**OR829678**	–	**This study**
*Perennicordycepscuboidea*	NBRC 103836	JN941721	JN943332	JN941420	AB972951	JN992455	AB972955	Schoch et al. (2012)
*Perennicordycepscuboidea*	NBRC 101740	JN941724	JN943331	JN941417	KF049684	JN992458	–	Schoch et al. (2012)
*Perennicordycepscuboidea*	TNS-F-18487	KF049609	–	KF049628	KF049683	–	–	Kepler et al. (2013)
*Perennicordycepscuboidea*	NBRC 101739	–	AB378668	AB378649	–	–	–	Ban et al. (2009)
*Perennicordycepselaphomyceticola*	NTUCC 17-022	–	MK840824	MK840813	MK839230	MK839221	MK839212	Yang et al. (2020)
*Perennicordycepselaphomyceticola*	MFLU 21-0262	OQ172101	OQ172064	OQ172032	OQ459718	OQ459747	OQ459792	Xiao et al. (2023)
*Perennicordycepselaphomyceticola*	MFLU 21-0263	OQ172102	OQ172065	OQ172033	OQ459719	OQ459748	OQ459793	Xiao et al. (2023)
*Perennicordycepselaphomyceticola*	MFLU 21-0264	OQ172103	OQ172067	OQ172035	OQ459720	OQ459749	OQ459794	Xiao et al. (2023)
*Perennicordycepselaphomyceticola*	MFLU 21-0266	OQ172112	OQ172068	OQ172036	OQ459732	OQ459760	OQ459806	Xiao et al. (2023)
***Perennicordycepselaphomyceticola***	**KUNCC23-16074**	**PP129613**	**OR878367**	**OR828243**	–	**OR829679**	–	**This study**
*Perennicordycepslutea*	KUMCC 3004	–	–	OQ474910	–	–	–	Xiao et al. (2023)
*Perennicordycepsparacuboidea*	NBRC 100942	JN941711	JN943337	JN941430	AB972954	JN992445	AB972958	Schoch et al. (2012)
*Perennicordycepsparacuboidea*	NBRC 101742	JN941710	JN943338	JN941431	KF049685	JN992444	KF049669	Schoch et al. (2012)
*Perennicordycepsprolifica*	NBRC 100744	JN941709	AB925942	JN941432	AB972952	JN992443	AB972956	Ban et al. (2015)
*Perennicordycepsprolifica*	NBRC 101750	JN941708	JN943340	JN941433	AB972953	JN992442	AB972957	Ban et al. (2015)
*Perennicordycepsprolifica*	TNS-F-18547	KF049613	KF049660	KF049632	KF049687	KF049649	KF049670	Kepler et al. (2013)
*Perennicordycepsprolifica*	NBRC 103839	JN941706	JN943342	JN941435	–	JN992440	–	Schoch et al. (2012)
*Perennicordycepsprolifica*	NBRC 103838	JN941707	JN943339	JN941434	–	JN992441	–	Schoch et al. (2012)
*Perennicordycepsprolifica*	TNS-F-18481	KF049612	KF049659	KF049631	KF049686	KF049648	–	Kepler et al. (2013)
*Perennicordycepsprolifica*	–	AB027324	–	AB027370	–	–	–	Nikoh and Fukatsu. (2000)
*Perennicordycepsryogamiensis*	NBRC 103842	JN941701	JN943345	JN941440	–	JN992435	–	Schoch et al. (2012)
*Perennicordycepsryogamiensis*	NBRC 101751	JN941703	JN943343	JN941438	KF049688	JN992437	–	Schoch et al. (2012)
*Pleurocordycepsagarica*	YHHPA1305^T^	KP276655	KP276651	–	KP276659	KP276663	KP276667	Wang et al. (2015a, b)
*Pleurocordycepsagarica*	YHCPA1303	KP276657	KP276653	–	KP276661	KP276665	KP276669	Wang et al. (2015a, b)
*Pleurocordycepsagarica*	YHCPA1307	KP276658	KP276654	–	KP276662	KP276666	KP276670	Wang et al. (2015a, b)
*Pleurocordycepsaurantiacus*	MFLUCC 17-2113^T^	MG136904	MG136916	MG136910	MG136874	MG136866	MG136870	Xiao et al. (2018)
*Pleurocordycepsaurantiacus*	MFLU 17-1393^T^	MG136907	MG136919	MG136913	MG136877	MG136868	MG136873	Xiao et al. (2018)
*Pleurocordycepsaurantiacus*	MFLU 21-0276	OQ172097	OQ172072	OQ172042	OQ459714	–	OQ459788	Xiao et al. (2023)
*Pleurocordycepsaurantiacus*	GACP 20-2306	OQ172098	OQ172069	OQ172041	OQ459715	–	OQ459789	Xiao et al. (2023)
*Pleurocordycepsformosus*	ARSEF 1424	KF049615	KF049661	KF049634	KF049689	KF049651	KF049671	Kepler et al. (2013)
*Pleurocordycepsheilongtanensis*	KUMCC 3008	OQ172111	OQ172091	OQ172063	OQ459731	OQ459759	OQ459805	Xiao et al. (2023)
*Pleurocordycepskanzashianus*	–	AB027325	AB027371	AB027371	–	–	–	Kepler et al. (2013)
*Pleurocordycepslanceolatus*	GACP 17-2004^T^	OQ172110	OQ172076	OQ172046	OQ459726	OQ459754	OQ459800	Xiao et al. (2023)
*Pleurocordycepslanceolatus*	GACP 17-2005^T^	OQ172109	OQ172077	OQ172047	OQ459727	OQ459755	OQ459801	Xiao et al. (2023)
*Pleurocordycepslianzhouensis*	HIMGD20918	KF226245	EU149921	KF226246	KF226248	KF226247	–	Zhang et al. (2007)
*Pleurocordycepslianzhouensis*	GIMYY9603	KF226249	EU149922	KF226250	KF226252	KF226251	–	Zhang et al. (2007)
*Pleurocordycepsmarginaliradians*	MFLUCC 17-2276	MG136909	MG136921	MG136915	MG136879	–	MG271930	Xiao et al. (2018)
*Pleurocordycepsmarginaliradians*	MFLU 17-1582	MG136908	MG136920	MG136914	MG136878	MG136869	MG271931	Xiao et al. (2018)
*Pleurocordycepsnipponicus*	BCC 1881	KF049618	–	KF049636	KF049692	–	KF049674	Kepler et al. (2013)
*Pleurocordycepsnipponicus*	NHJ 4268	KF049621	–	KF049639	KF049695	KF049654	KF049676	Kepler et al. (2013)
*Pleurocordycepsnipponicus*	BCC 2325	KF049622	KF049665	KF049640	KF049696	KF049655	KF049677	Kepler et al. (2013)
*Pleurocordycepsnipponicus*	BCC 18108	MF416624	KF049657	MF416569	MF416517	MF416676	MF416462	Kepler et al. (2013)
*Pleurocordycepsnipponicus*	NBRC 101408	JN941751	JN943303	JN941390	–	JN992485	–	Schoch et al. (2012)
*Pleurocordycepsnipponicus*	NBRC 101407	JN941752	JN943302	JN941389	–	JN992486	–	Schoch et al. (2012)
*Pleurocordycepsnipponicus*	NBRC 101406	JN941753	JN943301	JN941388	–	JN992487	–	Schoch et al. (2012)
*Pleurocordycepsnipponicus*	Cod-RE1202	MG031286	KX827757	MG031248	MG196133	MG196175	–	Sangdee et al. (2017)
*Pleurocordycepsnipponicus*	BCC 1682	KF049620	KF049664	KF049638	KF049694	–	–	Kepler et al. (2013)
*Pleurocordycepsnutansis*	MFLU 21-0275^T^	OQ172119	OQ172073	OQ172048	–	–	–	Xiao et al. (2023)
*Pleurocordycepsnutansis*	GACP 19-1906	OQ172117	OQ172079	OQ172049	–	–	–	Xiao et al. (2023)
*Pleurocordycepsonorei*	BRA: CR23902^T^	–	KU898841	–	–	–	–	Crous et al. (2017a)
*Pleurocordycepsonorei*	BRA: CR23904	–	KU898843	–	–	–	–	Crous et al. (2017a)
*Pleurocordycepsparvicapitata*	MFLU 21-0272	OQ172099	OQ172084	OQ172056	OQ459716	OQ459745	OQ459790	Xiao et al. (2023)
*Pleurocordycepsparvicapitata*	MFLU 21-0273	OQ172100	OQ172085	OQ172057	OQ459717	OQ459746	OQ459791	Xiao et al. (2023)
*Pleurocordycepsparvicapitata*	MFLU 21-0270	OQ172105	OQ172082	OQ172054	OQ459722	OQ459751	OQ459796	Xiao et al. (2023)
*Pleurocordycepsparvicapitata*	MFLU 21-0271^T^	OQ172106	OQ172083	OQ172055	OQ459723	OQ459752	OQ459797	Xiao et al. (2019)
***Pleurocordycepsparvicapitata***	**HKAS 131924**	**PP129615**	**OR878368**	**OR835990**	**OR880682**	**OR880686**	–	**This study**
***Pleurocordycepsparvicapitata***	**KUNCC23-16075**	**PP129616**	**OR878369**	**OR835991**	–	**OR880687**	–	**This study**
***Pleurocordycepsparvicapitata***	**HKAS 131925**	–	**OR878366**	**OR828241**	**OR880680**	**OR829677**	**OR880684**	**This study**
*Pleurocordycepsphaothaiensis*	BCC84551	–	MF959731	MF959735	MF959739	MF959743	–	Crous et al. (2017a)
*Pleurocordycepsphaothaiensis*	BCC84552	–	MF959732	MF959736	MF959740	MF959744	–	Crous et al. (2017a)
*Pleurocordycepsramosopulvinatus*	SU-65	–	–	DQ118742	DQ118753	DQ127244	–	Chaverri et al. (2005)
*Pleurocordycepsramosopulvinatus*	EFCC 5566	–	KF049658	KF049627	KF049682	KF049645	–	Kepler et al. (2013)
*Pleurocordycepsramosopulvinatus*	–	AB027326	AB027372	–	–	–	–	Nikoh and Fukatsu (2000)
*Pleurocordycepssinensis*	CN 80-2	HQ832887	HQ832884	HQ832886	HQ832890	HQ832888	HQ832889	Wang et al. (2012)
*Pleurocordycepssinensis*	GACP 20-2304	OQ172107	OQ172074	OQ172044	OQ459724	–	OQ459798	Xiao et al. (2023)
*Pleurocordycepssinensis*	GACP 20-2305	OQ172108	OQ172075	OQ172045	OQ459725	OQ459753	OQ459799	Xiao et al. (2023)
*Pleurocordycepssinensis*	MFLU 21-0267	OQ172121	OQ172081	OQ172051	OQ459741	OQ459767	OQ459813	Xiao et al. (2023)
*Pleurocordycepssinensis*	MFLU 21-0269	OQ172122	OQ172080	OQ172050	OQ459742	OQ459768	OQ459814	Xiao et al. (2023)
*Pleurocordycepssinensis*	GACP 19-2301	OQ172124	OQ172078	OQ172053	OQ459744	–	OQ459816	Xiao et al. (2023)
*Pleurocordycepssinensis*	GZU 20-0865	OQ172096	OQ172071	OQ172043	OQ459713	–	–	Xiao et al. (2023)
*Pleurocordycepssinensis*	HMAS 43720^T^	NR_119928	NG_042573	–	–	–	–	Wang et al. (2012)
*Pleurocordycepssinensis*	CGMCC 3.19069	MH454346	MH459160	–	–	–	–	Sun et al. (2019)
*Pleurocordycepssinensis*	–	–	HQ918290	–	–	–	–	Zhu et al. (2010)
*Pleurocordyceps* sp.	JB07.08.16_08	KF049616	KF049662	KF049635	KF049690	KF049652	KF049672	Kepler et al. (2013)
*Pleurocordyceps* sp.	JB07.08.17_07b	KF049617	–	–	KF049691	KF049653	KF049673	Kepler et al. (2013)
*Pleurocordyceps* sp.	BCC 2637	KF049619	KF049663	KF049637	KF049693	–	KF049675	Kepler et al. (2013)
*Pleurocordyceps* sp.	GIMCC 3.570	JX006097	JX006099	JX006098	JX006100	JX006101	–	Wang et al. (2020)
*Pleurocordyceps* sp.	NBRC 109990	–	–	AB925968	–	–	–	Wang et al. (2020)
*Pleurocordyceps* sp.	NBRC 110224	–	AB925931	AB925969	–	–	–	Unpublished
*Pleurocordyceps* sp.	NBRC 109987	–	AB925947	AB925983	–	–	–	Unpublished
*Pleurocordyceps* sp.	NBRC 109988	–	AB925948	AB925984	–	–	–	Unpublished
*Pleurocordyceps* sp.	–	HM135166	HM135164	HM135165	–	–	–	Wang et al. (2020)
*Pleurocordyceps* sp.	NBRC 110223	–	AB925930	–	–	–	–	Unpublished
*Pleurocordycepsvitellina*	KUMCC 3006	–	OQ172089	OQ172061	OQ459729	OQ459757	OQ459803	Xiao et al. (2023)
*Pleurocordycepsvitellina*	KUMCC 3007	–	OQ172090	OQ172062	OQ459730	OQ459758	OQ459804	Xiao et al. (2023)
*Pleurocordycepsyunnanensis*	YHH PY1006^T^	–	KF977849	–	KF977851	KF977853	KF977855	Wang et al. (2015a, b)
***Pleurocordycepsyunnanensis***	**HAKS 131922**	**PP129614**	–	**OR828244**	**OR880681**	**OR829680**	–	**This study**
*Pleurocordycepsyunnanensis*	YHC PY1005	–	KF977848	–	KF977850	KF977852	KF977854	Wang et al. (2015a, b)
*Polycephalomycesalbiramus*	GACP 21-XS08^T^	OQ172115	OQ172092	OQ172037	OQ459735	OQ459761	OQ459807	Xiao et al. (2023)
*Polycephalomycesalbiramus*	GACPCC 21-XS08^T^	OQ172116	OQ172093	OQ172038	OQ459736	OQ459762	OQ459808	Xiao et al. (2023)
*Polycephalomycesformosus*	NBRC 100686	MN586821	MN586830	MN586839	MN598054	MN598045	MN598061	Wang et al. (2020)
*Polycephalomycesformosus*	NBRC 100687	MN586822	MN586831	MN586840	MN598055	MN598046	MN598062	Wang et al. (2020)
*Polycephalomycesformosus*	NBRC 103843	MN586823	MN586832	MN586841	MN598056	MN598047	MN598063	Wang et al. (2020)
*Polycephalomycesformosus*	NBRC 109993^T^	MN586824	MN586833	MN586842	MN598057	MN598048	MN598064	Wang et al. (2021)
*Polycephalomycesformosus*	NBRC 109994	MN586825	MN586834	MN586843	MN598058	MN598049	MN598065	Wang et al. (2020)
*Polycephalomycesformosus*	GACP 21-WFKQ03	OQ172113	OQ172094	OQ172039	–	–	–	Xiao et al. (2023)
*Polycephalomycesformosus*	GACP 21-WFKQ04	OQ172114	OQ172095	OQ172040	–	–	–	Xiao et al. (2023)
*Polycephalomycesramosus*	NBRC 101760	MN586818	MN586827	MN586836	MN598051	MN598042	MN598060	Wang et al. (2020)
*Polycephalomycesramosus*	NBRC 109984	MN586819	MN586828	MN586837	MN598052	MN598043	–	Wang et al. (2020)
*Polycephalomycesramosus*	NBRC 109985	MN586820	MN586829	MN586838	MN598053	MN598044	–	Wang et al. (2020)
*Polycephalomycesramosus*	MFLU 18-0162^T^	MK863043	MK863250	MK863050	MK860188	–	–	Unpublished
*Polycephalomycesramosus*	NBRC 109983	–	AB925946	AB925982	–	–	–	Unpublished
*Polycephalomycesramosus*	RUTPP	–	–	AY259543	–	–	–	Bischof et al. (2003)
*Polycephalomycesramosus*	RCEF 6016	–	KC782530	–	–	–	–	Crous et al. (2017a)
***Polycephalomycestengchongensis***	**HKAS 131923^T^**	**PP129612**	**OR878365**	**OR828240**	–	**OR829676**	**OR880685**	**This study**
*Polycephalomycestomentosus*	BL 4	KF049623	KF049666	KF049641	KF049697	KF049656	KF049678	Kepler et al. (2013)
*Tolypocladiumophioglossoides*	NBRC 100998	JN941735	JN943319	JN941406	AB968602	JN992469	AB968563	Ban et al. (2015)
*Tolypocladiumophioglossoides*	NBRC 106330	JN941734	JN943321	JN941407	AB968603	JN992468	AB968564	Ban et al. (2015)

**Abbreviations: ARSEF**: Agricultural Research Service Entomopathogenic Fungus Collection, USDA, USA; **BCC**: BIOTEC Culture Collection, Klong Luang, Thailand; **EFCC**: Entomopathogenic Fungal Culture Collection, Chuncheon, Korea; **GZUH/GACP**: Herbarium of Guizhou University, China; **GZUIFR**: Institute of Fungal Resources of Guizhou University, China; **HKAS**: Kunming Institute of Botany, Academia Sinica, China; **NBRC**: Biological Resource Center, the National Institute of Technology and Evaluation, Japan; **NHJ**: Nigel Hywel-Jones personal collection, Thailand; **MFLU**: Mae Fah Luang University, Thailand; **KUNCC**: Kunming Institute of Botany Culture Collection, China; **NTUCC**: National Taiwan University Cancer Center, China; **PDD**: Dried specimens have been deposited in the New Zealand Fungarium, New Zealand; **CGMC**: China General Microbiological Culture Collection Center, China; **JB**: Joseph Bischoff, personal collection, Britain; **TNS**: National Museum of Science and Nature, Tsukuba, Japan; **YHH/YHC**: Yunnan Herbal Herbarium, China; **RTUPP**: Rutgers Mycological Herbarium.

## ﻿Results

### ﻿Phylogenetic analyses

The phylogenetic analysis was constructed using sequence data from six loci, representing 112 Polycephalomycetaceae taxa. The alignment comprised 5142 base pair (bp) characters, including gaps (1026 bp for SSU, 602 bp for ITS, 845 bp for LSU, 901 bp for *tef-1α*, 711 bp for *rpb1*, and 1057 bp for *rpb2*). Of these, 3648 characters were constant, 1310 variable characters parsimony-uninformative and 1936 characters parsimony-informative. The likelihood of the best-scoring ML tree was -26079.662.

**Figure 1.**
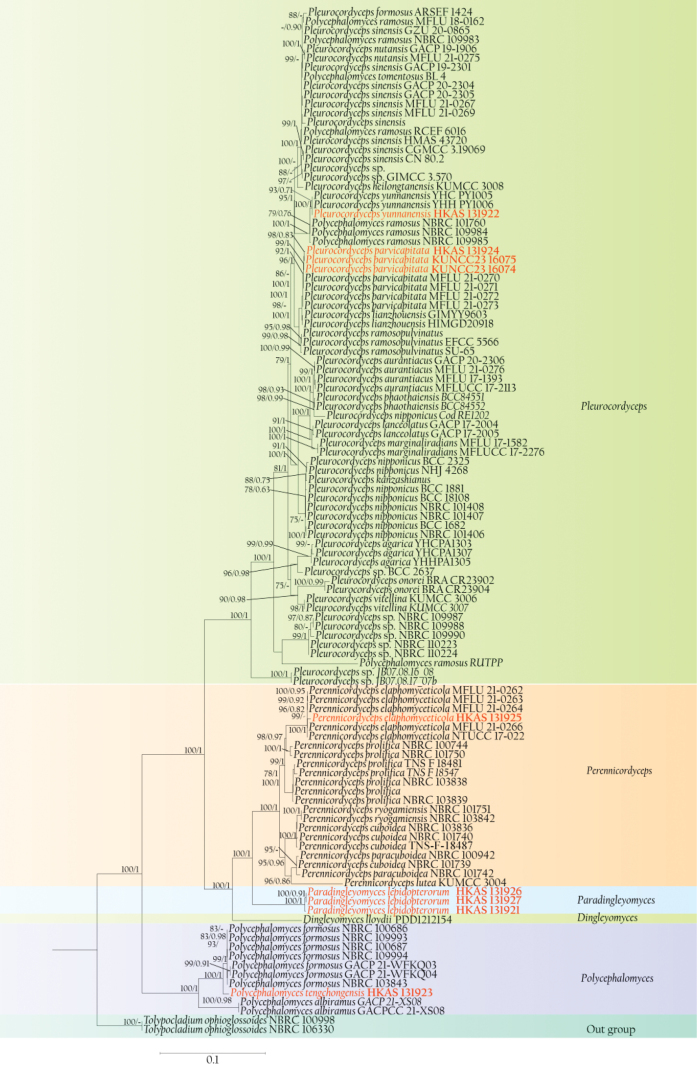
Phylogenetic tree of Polycephalomycetaceae based on a concatenated data matrix of SSU, ITS, LSU, *tef-1α*, *rpb1*, and *rpb2*. Bootstrap values greater than or equal to 75% and Bayesian posterior probabilities greater than or equal to 0.80 are shown at the respective nodes. Newly added taxa from this study are highlighted in red.

In the phylogenetic analyses (Fig. 1), two strains of *Tolypocladiumophioglossoides* (NBRC 100998 and NBRC 106330) were used as outgroup taxa. In the multi-locus phylogenetic tree (Fig. 1), our specimens were distributed across four clades, representing one new genus, one new species and three known species. The strain of *Paradingleyomyceslepidopterorum* (HKAS 131926, HKAS 131927 and HKAS 131921) formed a distinct clade, positioned between *Dingleyomyces* and *Perennicordyceps*, with maximum statistical support (MLBS = 100%, BIPP = 1.00). *Polycephalomycestengchongensis* (HKAS 131923) branches off from *Polycephalomycesformosus* with significant support (MLBS = 100%, BIPP = 1.00). *Pleurocordycepsyunnanensis* (HKAS 131922) groups with *Pleurocordycepsyunnanensis* (YHH PY1006 and YHC PY1005) with strong support (MLBS = 84%, BIPP = 0.99). *Pleurocordycepsparvicapitata* (HKAS 131924), along with its isolate KUNCC23-16075 and the isolate KUNCC23-16074 (which was isolated from the sclerotium of specimen HKAS 131925) clusters with *Pleurocordycepsparvicapitata* with adequate support (MLBS = 90%, BIPP = 1.00). *Perennicordycepselaphomyceticola* (HKAS 131925), which represents the host of *Pleurocordycepsparvicapitata* phylogenetically clusters with *Perennicordycepselaphomyceticola* (MFLU 21-0262, MFLU 21-0263, MFLU 21-0264, MFLU 21-0266, and NTUCC 17-022) with strong support (MLBS = 100%, BIPP = 1.00).

### ﻿Taxonomy

#### ﻿ Paradingleyomyces

Taxon classificationFungiHypocrealesPolycephalomycetaceae

Y. Wang tris & T. C. Wen gen. nov.

E8B4C64C-BD50-5351-8038-26A28D92A89B

Index Fungorum: IF901540

##### Etymology.

Morphologically resembling the genus *Dingleyomyces*.

##### Type species.

*Paradingleyomyceslepidopterorum* Y. Wang tris & T. C. Wen, sp. nov.

##### Description.

Parasitic on Ophiocordycepscf.cochlidiicola. Sexual morph: Stroma absent. ***Perithecia*** forming from white subiculum covering stromata of Ophiocordycepscf.cochlidiicola, superficial, scattered, brown, ovoid or ellipsoidal. ***Asci*** cylindrical with a thickened cap, attenuated toward the base. ***Ascospores*** filiform, hyaline, disarticulating into many cylindrical secondary ascospores at maturity. ***Secondary ascospores*** cylindrical, aseptate, smooth-walled, with truncated ends. Asexual morph: Undetermined.

##### Notes.

Both *Paradingleyomyces* and *Dingleyomyces* are monotypic genera and *s*hare similar morphological characteristics, including the formation of superficial perithecia on a white subiculum, cylindrical asci with thickened caps, and filiform ascospores that disarticulate into cylindrical secondary ascospores. Additionally, the type species of both genera occur as hyperparasites on *Ophiocordyceps* species (Johnston and Park 2023). However, multigene phylogenetic analysis revealed that these two genera exhibit a paraphyletic relationship, indicating they are not congeneric. *Paradingleyomyces* can be easily distinguished from *Perennicordyceps* by its reduced stromata, whereas *Perennicordyceps* features cylindrical to clavate, branched stromata with prominent rhizomorphs immersed in the substrate, and perithecia forming from the middle to upper parts of the stromata (Ban et al. 2009; Xiao et al. 2019, 2023).

#### ﻿ Paradingleyomyces lepidopterorum

Taxon classificationFungiHypocrealesPolycephalomycetaceae

Y. Wang tris & T. C. Wen sp. nov.

A51F665D-D145-5CC8-98E0-4356E58AE8B9

Index Fungorum: IF901541

Fig. 2

##### Etymology.

This epithet is named after the order of its primary host: Lepidoptera.

##### Description.

Parasitic on Ophiocordycepscf.cochlidiicola. ***Stromata*** of host fungus are 55–180 mm in length, 1–3 mm in width, multiple, unbranched, brown at base becoming off-white toward the apex, fibrous, narrowly cylindrical to filiform. Sexual morph: ***Subiculum*** white, cottony, covering the stromata of host fungus. ***Perithecia*** 240–690 × 110–360 μm (x̄ = 430 × 228 μm, n = 25), emerging from subiculum, superficial, scattered or dense, flesh-colored, ovoid or ellipsoidal. ***Asci*** 150–400 × 3–8 μm (x̄ = 289 × 5 μm, n = 30), cylindrical, hyaline, with an apical cap. ***Apical cap*** 3–5 × 1–4 μm (x̄ = 3.8 × 2.3 μm, n = 40), hemispherical. ***Ascospores*** filiform, multiseptate, breaking into many secondary ascospores at maturity. ***Secondary ascospores*** 2–4 × 0.5–1 μm (x̄ = 2.5 × 0.9 μm, n = 50), hyaline, aseptate, smooth-walled, cylindrical with truncated ends. Asexual morph: Undetermined.

##### Distribution.

China: Yunnan Province.

##### Material examined.

***Holotype***: China • Yunnan Province, Tengchong County, Houqiao Town; 5 Nov. 2022; Collected by Yi Wang; Parasitic on the stromata of Perennicordycepscf.elaphomyceticola; GYY543H (HKAS 131926) • ***Paratypes***: *ibid*; GYY543Z (HKAS131927), TC327 (HKAS 131921).

##### Notes.

**Figure 2.**
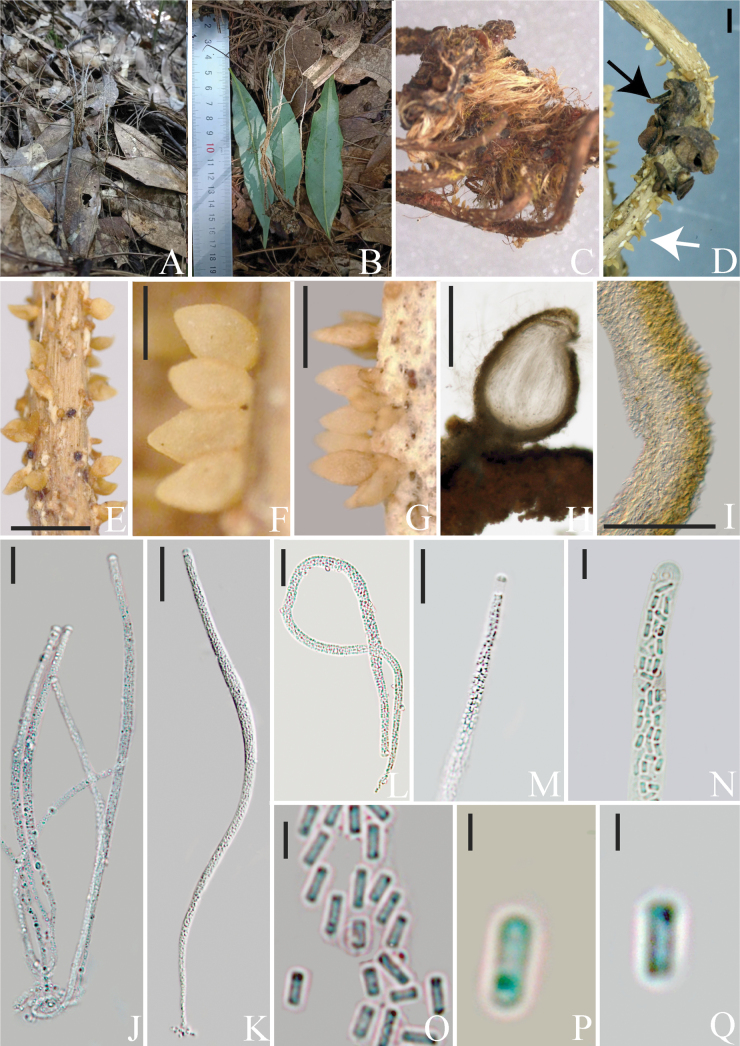
*Paradingleyomyceslepidopterorum* (HKAS 131926, **holotype**) **A** habitat **B** stromata growing from the host **C** host **D** perithecia of *Pa.lepidopterorum* (white arrow) and Ophiocordycepscf.cochlidiicola (black arrow) **E–G** perithecia forming on white subiculum **H** vertical section of perithecium. I Peridium **J–L** asci **M, N** apical cap of asci **O–Q** secondary ascospores. Scale bars: 1 mm (**E–G**); 500 μm (**H**); 100 μm (**I**); 50 μm (**J, K**); 25 μm (**L**); 20 μm (**M, N**); 5 μm (**O–Q**).

*Paradingleyomyceslepidopterorum* lives as a hyperparasite on the remnant stromata of Ophiocordycepscf.cochlidiicola. The aging stromata of the host fungus become covered with the perithecia of the hyperparasitic fungus, which closely resemble those of the host. However, the key distinguishing feature is that the hyperparasitic perithecia are flesh-colored and grow on a white subiculum, whereas the host’s perithecia are dark brown and directly connected to the stroma (Fig. 2D). *Paradingleyomyceslepidopterorum* and *Dingleyomyceslloydii* are morphologically very similar, but they can be easily distinguished from *Perennicordyceps* species by the presence of a white subiculum from which the perithecia arise (Table 2). In contrast, *Perennicordyceps* is characterized by cylindrical to clavate, branching stromata with the host and rhizomorphs embedded in the substrate. *Dingleyomyceslloydii* produce crown-like perithecia on the stromata of *Ophiocordycepshauturu* and *O.robertsii*, while the perithecia of *Pa.lepidopterorum* sporadically form on the stromata of O.cf.cochlidiicola.

**Table 2.** Morphological comparison between sexual species in *Paradingleyomyces*, *Perennicordyceps*, and *Dingleyomyces*.

Species	Host	Stromata (mm)	Perithecia (µm)	Asci (µm)	Apical cap (µm)	Secondary ascospores (µm)	References
*Dingleyomyceslloydii*	*Ophiocordycepshauturu, Ophiocordycepsrobertsii*	Reduced to white subiculum, ﬂat, thin, irregular plates, often obscured by the perithecia, white or yellowish	300–950 × 300–650, superficial, ovoid, growing in small groups on white subiculum	200–450 × 6–10	2–3 diameter, 4 thickness	1.5–3 × 1–1.5	Dingley (1953); Mains (1958); Johnston and Park. (2023)
*Perennicordycepselaphomyceticola*	*Elaphomyces* sp.	20–100 × 0.1–0.5, cylindrical, the colours vary from dark brown, titian red, brownish orange, yellow to pale	430–600 × 255–300, superficial, ovoid to ellipsoid, yellow when mature, pale when immature	365–420 × 5.0–7.6	2–3.5 × 3.3–5.2	1.5–3.2 × 1.4–2.3	Xiao et al. (2023)
*Pe.cuboidea*	Beetle larva or other *Cordyceps*	32–181 × 3–74, cylindrical, ochre yellow	400–500 × 250–330, superficial, lemon-shaped, glabrate	250–570 in length	3–5 in thickness	1.5–3 × 1–1.5	Ban et al. (2009)
*Pe.prolifica*	Cicada nymph	70.9–140.0× 0.8–2.2, thin cylindrical, brown	320–530 × 200–340, superficial, ovoid or ellipsoidal, grayish brown	430–650 in length	3–5 in thickness	2–3 × 1–2	Kobayasi and Shimizu (1963)
***Pa.lepidopterorum***	**Ophiocordycepscf.cochlidiicola**	**Reduced to white subiculum**	**240–690 × 110–360, superficial, ovoid to ellipsoidal, brown**	**150–400 × 3–8**	**3–5 × 1–4**	**2–4 × 0.5–1**	**This study**
*Pe.paracuboidea*	Coleopteran larva	3.2–38.4 × 0.3–1.7, cylindrical	400–600 × 290–400, superficial, lemon-shaped, pale orangish brown	225–400 in length	3–6.3 in thickness	1.3–2.5 × 1–2	Ban et al. (2009)
*Pe.ryogamiensis*	Coleopteran larva	12–13 × 0.5, cylindrical, white, palely darkened, glabrate at the base	320–430 × 200–230, superficial, ovoid	450–610 in length	3.8–5 in thickness	2.5–5 × 1.5–2	Ban et al. (2009)

#### ﻿ Polycephalomyces tengchongensis

Taxon classificationFungiHypocrealesPolycephalomycetaceae

Y. Wang tris & T. C. Wen sp. nov.

0C0A9D1F-B6A5-5D4F-9CE8-0B55C3D94BF8

Index Fungorum: IF901449

Fig. 3

##### Etymology.

Named after the location where the type specimen was found, Tengchong County, Yunnan Province.

##### Description.

Parasitic on Perennicordycepscf.elaphomyceticola. Sexual morph: Undetermined. Asexual morph: ***Synnemata*** 18.7 mm long, 1–2 mm wide, cylindrical, white, growing in small group on stromata of Perennicordycepscf.elaphomyceticola. ***Fertile parts*** yellowish, with conidial mass forming on middle part of synnemata. ***Phialides*** dimorphic. ***α-phialides*** 9–20 × 1–2 μm (x̄ = 12.3 × 1.2 μm, n = 45), phialidic, subulate, hyaline, smooth-walled, arranged in a parallel palisade-like layer around the fertile part. ***α-conidia*** 1–3 μm (x̄ = 2 μm, n = 45), globose, hyaline.

**Figure 3.**
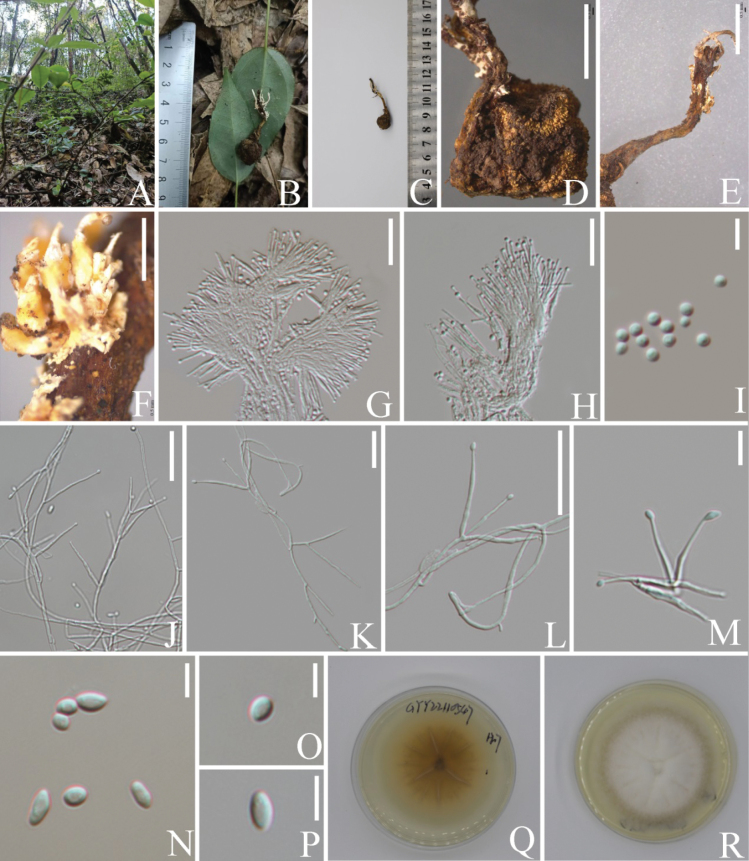
*Polycephalomycestengchongensis* (**B–I** from HKAS 131923, **J–R** from KUNCC23-16073) **A** habitat **B–F** infected Perennicordycepscf.elaphomyceticola showing synnemata of parasites **G, H** α-phialides **I** α-conidia **J–M** β-phialides **N–P** β-conidia **Q, R** reverse and front view of culture on PDA. Scale bars: 5 mm (**D–F**); 20 μm (**G, H**); 50 μm (**J–L**); 10 μm (**M**); 5 μm (**I, N–P**).

##### Culture characters.

Colonies on PDA attaining a diam. of 28–31 mm in 14 days at 25 °C, white, leathery, radially striate, reverse dark brown and turns pale outward. ***β-phialides*** 18–44 × 1–3 μm (x̄ = 26.7 × 1.2 μm, n = 30), phialidic, lanceolate, smooth-walled. ***β-conidia*** 3–7 × 1.5–3 μm (x̄ = 3.9 × 2.2 μm, n = 45), ellipsoidal to broadly fusiform, hyaline, aseptate, smooth-walled.

##### Material examined.

China • Yunnan Province, Tengchong County, Houqiao Town; 5 Nov. 2022; Collected by Yi Wang; Parasitic on the stromata of Perennicordycepscf.elaphomyceticola; GYY547 (HKAS 131923, ex-holotype living culture: KUNCC23-16073).

##### Notes.

The newly described species *Polycephalomycestengchongensis* is closely related to *Po.formosus* with strong support (MLBS = 100%, MIPP = 1.00, Fig. 1). However, *Polycephalomycestengchongensis* is distinct from *Po.formosus* in several aspects. It parasitizes Perennicordycepscf.elaphomyceticola and produces synnemata without well-defined stipe and a fertile head but features dimorphic phialides and conidia. In contrast, *Po.formosus* has stipitate synnemata with a fertile head at the tip and produces only one type of phialides and conidia (Xiao et al. 2023).

A comparison of nucleotide sequences between *Po.tengchongensis* and the ex-type of strain of *Po.formosus* (NBRC 109993) revealed 1% differences (6/584 bp) including three gaps in the ITS region, 0.3% (3/774 bp) differences including one gap in the LSU region, 2.3% differences (16/684 bp) including three gaps in the *rpb1* region and 1.6% differences (15/891bp) in the *rpb2* region. Collectively, the differences both in phenotypic profiles and nucleotides sequences support the establishment of *Polycephalomycestengchongensis* as a new species.

#### ﻿ Pleurocordyceps parvicapitata

Taxon classificationFungiHypocrealesPolycephalomycetaceae

Y.P. Xiao, T.C. Wen & K.D. Hyde, in Xiao et al. Fungal Diversity 120: 50 (2023)

5B807045-3E54-53FC-BD9A-2368A560ED03

Index Fungorum: IF559473

Figs 4 , 5

##### Description.

**Figure 4.**
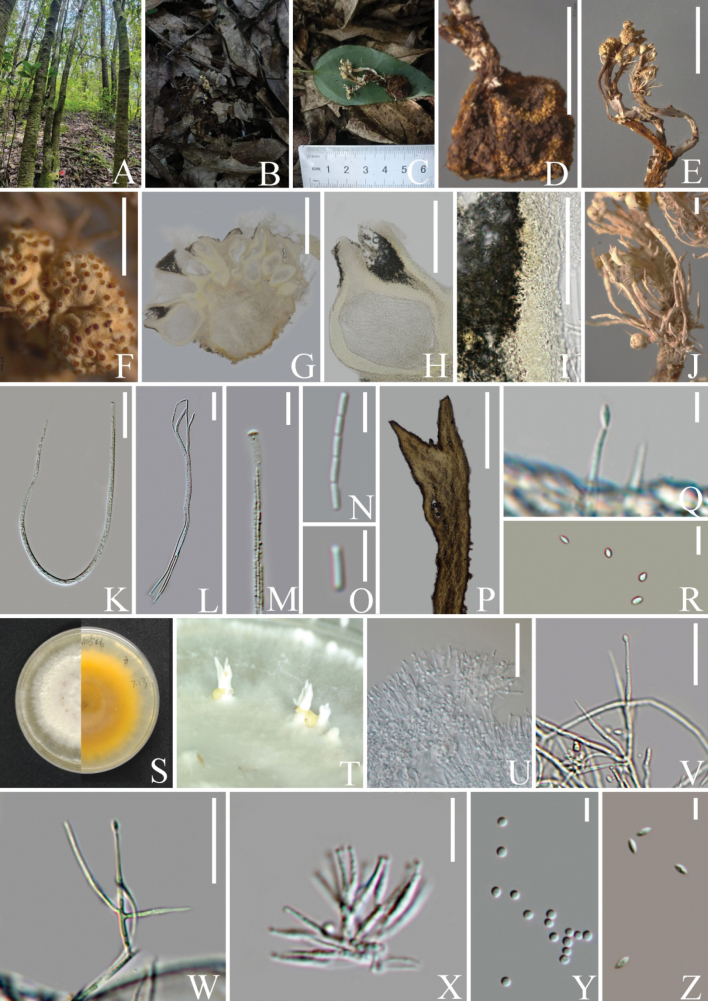
*Pleurocordycepsparvicapitata* (**B–R** from HKAS 131924 **S–Z** from KUNCC23-16075) **A** habitat **B, C** stromata emerging from host **D** host (*Elaphomyces* sp.) **E** fertile cushions on stromata **F** enlargement of fertile cushion**G** cross-section through fertile cushion**H** perithecium **I** peridium **J, P** synnemata on stromata **K** asci **L** part of asci **M** ascus cap **N, O** secondary ascospores **Q, V, W** β-phialides **S** front and reverse view of culture on PDA**T** synnemata on culture **U, X** α-phialides **Y** α-conidia **Z** β-conidia. Scale Bars: 5 mm (**E**); 1 mm (**D, F**); 500 µm (**G, J, P**); 250 µm (**H**); 100 µm (**I**); 50 µm (**K, L**); 20 µm (**M, N, V, U, W**); 10 µm (**X**); 5 µm (**O, Q, R, Y, Z**).

**Figure 5.**
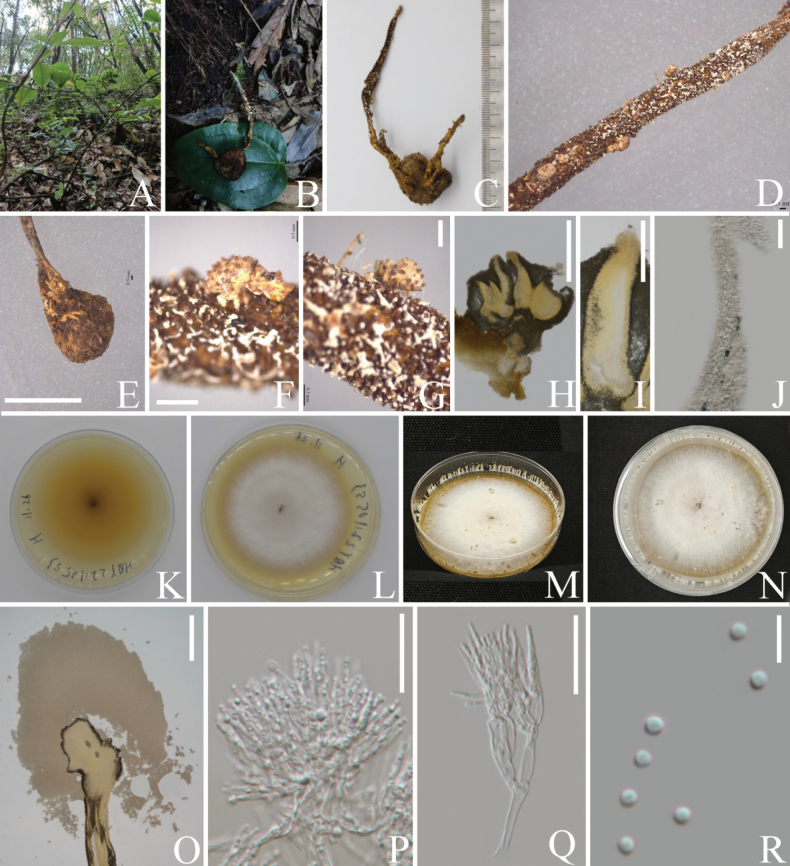
*Pleurocordycepsparvicapitata* (HKAS 131925) **A** habitat **B, C, E** host (*Perennicordycepselaphomyceticola*) **D, F, G** fertile cushions growing on stromata of *Pe.elaphomyceticola***H** cross-section through fertile cushion**I** perithecium **J** peridium **L** reverse and front view of culture on PDA after incubation for 14 days **M, N** front view of culture on PDA after incubation for 30 days **O** synnemata **P** conidiophores **Q** phialides **R** conidia. Scale bars: 1 mm (**E–G**); 500 µm (**H, I, O**); 20 µm (**J, P, Q**); 5 µm (**R**).

Parasitic on *Elaphomyces* sp. (Fig. 4). The host 6–10 mm in diam., dark brown or brown, spherical, hard, and rough on the surface. Sexual morph: ***Stromata*** 18–21 mm long, 1–2 mm wide, brown, multiple, fibrous. ***Stipe*** 8–15 mm long, 0.5–1 mm wide, brown, cylindrical, terminally or laterally carrying fertile cushions. ***Fertile cushions*** 0.5–1 mm in height, 1–2 mm in width, pale yellow to yellow, hemispherical. ***Perithecia*** 160–530 × 100–305 µm (x̄ = 306 × 179 µm, n = 20), immersed, crowded, ovoid to obpyriform, ostiolate. ***Peridium*** 15–40 µm (x̄ = 25 µm, n = 20) wide, three-layered, comprised of hyaline to pale yellow cells of ***textura intricate*** at outermost layer to ***textura angularis*** at middle layer to *t****extura prismatica*** at inner layer. ***Asci*** 190–380 × 3–5 µm (x̄ = 252 × 3.9 µm, n = 50), cylindrical, with thickened apex. ***Apical cap*** 1–2 × 2.5–4 μm (x̄ = 1.7 × 3.4 µm, n = 60), hyaline. ***Ascospores*** filiform, multiseptate, hyaline, breaking into many secondary ascospores at maturity. ***Secondary ascospores*** 2–8 × 0.5–1 µm (x̄ = 5.1 × 0.9 µm, n = 50), cylindrical, aseptate, straight, smooth-walled. Asexual morph: ***Synnemata*** cylindrical, off-white, gregarious, unbranched, occurring in close proximity to the fertile cushions. ***β-phialides*** up to 16 µm in length, 2 µm in width, cylindrical, attenuate toward the apex, phialidic, hyaline, smooth-walled. ***β-conidia*** 3–5 × 1–2 µm (x̄ = 3.8 × 1.7 µm, n = 20), fusiform, hyaline, aseptate. Additionally, *Pleurocordycepsparvicapitata* parasitic on *Perennicordycepselaphomyceticola* was found in proximity to the one on *Elaphomyces* sp. (Fig. 5). Sexual morph: ***Stromata*** not observed. ***Fertile cushion*** 0.5–1 mm long, 1–2 mm wide, directly growing on stromata of *Pe.elaphomyceticola*, pale yellow to yellow, surface wrinkle, rough due to the protruding perithecia. ***Perithecia*** 440–560 ×115–250 μm (x̄ = 505 × 170 µm, n = 15) ovoid to obpyriform, immersed, gregarious. ***Peridium*** 15–42 µm (x̄ = 25 µm, n = 20) wide, three-layered, comprised of hyaline to pale yellow cells of ***textura intricate*** at outermost layer to ***textura angularis*** at middle layer to ***textura prismatica*** at inner layer. Asci and ascospores were not observed due to the specimen being immature.

##### Culture characters.

Colonies isolated from *Elaphomyces* sp. and *Perennicordycepselaphomyceticola* present similar characteristics. Colonies on PDA attaining 41–45 mm in diam. after incubation at 25 °C for 14 days, white, leathery, reverse grayish yellow. ***Synnemata*** emerging from margin of colony in annular distributions, 1–3 mm long, 1–2.5 mm wide, white, erected, apically branched. ***Conidial mass*** gathers at middle part or tip of synnemata, pale yellow, waterish. ***Phialides*** two-typed. ***α-phialides*** 10–28 × 1–2 µm (x̄ = 15.7 × 1.5 µm, n = 50), hyaline, smooth-walled, subculate, caespitose, palisade-like. ***α-conidia*** 1–2 µm (x̄ = 1.7 µm, n = 45), one-celled, hyaline, smooth-walled, globose. ***β-phialides*** 6–8 × 0.5–1 µm (x̄ = 7.9 × 1.1 µm, n = 20), arising from hypha, solitary, lanceolate, hyaline, smooth-walled. ***β-conidia*** 2–6 × 1–2 µm (x̄ = 3.8 × 0.8 μm, n = 35), fusiform, hyaline, aseptate, smooth-walled, asymmetrical.

##### Material examined.

China • Yunnan Province, Tengchong County, Houqiao Town; 5 Nov. 2022; Collected by Yi Wang; Parasitic on *Elaphomyces* sp. buried in soil; GYY546 (HKAS 131924, living culture: KUNCC23-16075) • *ibid*; Parasitic on *Perennicordycepselaphomyceticola*; 5 Nov. 2022; Collected by Yi Wang; GYY553 (HKAS 131925, living culture: KUNCC23-16074).

##### Notes.

*Pleurocordycepsparvicapitata*, parasitic on *Elaphomyces* sp. and *Perennicordycepselaphomyceticola*, was originally described by Xiao et al. (2023) based on specimens collected from Dadugang County, Xishuangbanna, Yunnan Province, China. The specimen associated with *Elaphomyces* sp. produces pale yellow to yellow, wrinkled fertile cushions that are laterally or terminally attached to stromata, along with cylindrical asci, filiform, disarticulating ascospores and cylindrical, smooth-walled secondary ascospores. In this study, we collected a specimen displaying the typical characteristics of *Pl.parvicapitata* from Tengchong County, Yunnan Province. Importantly, Xiao et al. (2023) described *Pl.parvicapitata* as having one type of phialides and conidia from dry specimen. In contrast, we examined the asexual morph from both our specimens and its pure culture, observing dimorphic phialides and conidia. Additionally, the specimen associated with *Perennicordycepselaphomyceticola* was previously known only from its asexual morphs (Xiao et al. 2023), where the species was described as having pulvinate, yellowish conidiomata with one-type of phialides and conidia on the stromata of *Pe.elaphomyceticola*. In this study, we collected a sexual specimen from Tengchong County, Yunnan Province and its fertile cushion was similar to *Pl.parvicapitata* found on *Elaphomyces* sp. (Xiao et al. 2023). This is the first report of the sexual morph of *Pl.parvicapitata* on *Pe.elaphomyceticola*, which differs from previously described sexual morphs in that it directly forms a fertile cushion on the substrate. We have also supplemented this species with a pure culture which can be used for further research. These findings provide deeper insights into the morphological traits of *Pl.parvicapitata*.

#### ﻿ Pleurocordyceps yunnanensis

Taxon classificationFungiHypocrealesPolycephalomycetaceae

(Hong Yu bis, Y.B. Wang & Y.D. Dai) Y.H. Wang, S. Ban, W.J. Wang, Yi Li, Ke Wang, P.M. Kirk & Y.J. Yao, in Wang et al. Journal of Systematics and Evolution 59(5): 1076 (2021)

0A3FF45D-43C8-54B3-8313-2BCEEA325AC7

Index Fungorum: IF570681

Fig. 6

##### Description.

**Figure 6.**
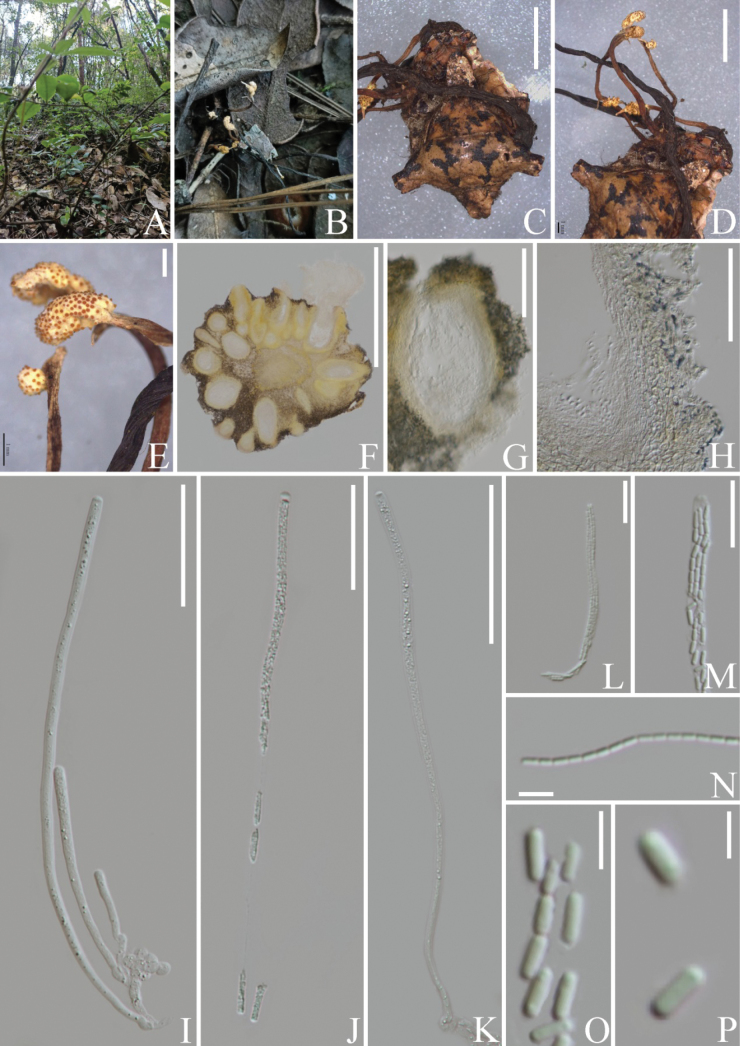
*Pleurocordycepsyunnanensis* (HAKS 131922) **A** habitat **B–D** stromata of *Pl.yunnanensis* growing from the host (*Ophiocordycepsnutans*) **E** fertile head**F** cross-section through fertile head**G** perithecium **H** peridium **I–K** asci **L, M** part of asci **N** part of ascospore **O, P** secondary ascospores. Scale bars: 5 mm (**C, D**); 1 mm (**E**); 100 µm (**F, G**); 50 µm (**H–K**); 20 µm (**L, M**); 5 µm (**N–P**).

Parasitic on *Ophiocordycepsnutans* (Ophiocordycipitaceae, Hypocreales) (Fig. 6). Sexual morph: ***Stromata*** 12–25 mm long, 0.5–1 mm wide, fibrous, brown, multiple, unbranched. ***Stipes*** 5–11 mm long, ca. 0.5 mm wide, brown to pale brown. ***Fertile head*** 1–2.5 mm long, 0.7–1.3 mm wide, yellowish to yellow, capitate, rough. ***Perithecia*** 160–390 × 55–170 μm (x̄ = 269 × 115 µm, n = 20), immersed, crowded, ovoid to obpyriform, ostiolate yellow, thick-walled. ***Peridium*** 14–46 µm (x̄ = 32 µm, n = 25) wide, three-layered, comprised of hyaline to yellowish cells of ***textura prismatica*** at outer layer to ***textura angularis*** at middle layer to ***textura porrecta*** at inner layer. ***Asci*** 95–235 × 3–6 µm (x̄ = 172 × 5 µm, n = 40), 8-spored, with thickened cap. ***Ascospores*** filiform, hyaline, multiseptate, disarticulating into many secondary ascospores at maturity. ***Secondary ascospores*** 2.5–5 × 1–2 µm (x̄ = 3.9 × 1.3 µm, n = 40), cylindrical, aseptate, hyaline, smooth-walled. Asexual morphs: see Wang et al. (2015a).

##### Material examined.

China • Yunnan Province, Kunming City, the Wild Duck Lake Forest Park; 17 Sep. 2023; Collected by Yi Wang; Parasitic on *Ophiocordycepsnutans*; YYH13 (HAKS 131922).

##### Notes.

The asexual morph of *Polycephalomycesyunnanensis* was first described by Wang et al. (2015a) from *Ophiocordycepsnutans* in Wild Duck Lake Forest Park, Kunming, Yunnan Province. This species was later transferred to *Pleurocordyceps* by Wang et al. (2021) based on molecular phylogenetic analyses. In this study, a sexual polycephalomyces-like fungus growing on *O.nutans* was obtained from the same location as the type specimen (Fig. 6). Phylogenetic analysis revealed that it groups with strains of *Pleurocordycepsyunnanensis* with strong support (Fig. 1). Therefore, we introduce our specimen as the new sexual morph of *Pl.yunnanensis*.

## ﻿Discussion

Polycephalomycetaceae was introduced by Xiao et al. (2023) to encompass the genera *Pleurocordyceps*, *Perennicordyceps*, and *Polycephalomyces*. The monophyletic nature of these three genera has been confirmed through numerous phylogenetic studies (Tian et al. 2010; Wang et al. 2012; Kepler et al. 2013; Wang et al.2015a; Zhong et al. 2016; Crous et al. 2017a; Sobczak etal. 2017; Poinar and Vega 2020; Xiao et al. 2018, 2023). Johnston and Park (2023) introduced a new genus, *Dingleyomyces*, into Polycephalomycetaceae. *Dingleyomyces* is a monotypic genus, and typified by *Dingleyomyceslloydii*, a species that is hyperparasitic on *Ophiocordycepshauturu* from New Zealand. *Dingleyomyceslloydii* was placed in a distant clade branching off from *Perennicordyceps* (Johnston and Park 2023). In this study, we introduce a new genus, *Paradingleyomyces* to accommodate *Pa.lepidopterorum* which forms a distinct clade nested between *Perennicordyceps* and *Dingleyomyces* (Fig. 1).

**Figure 7.**
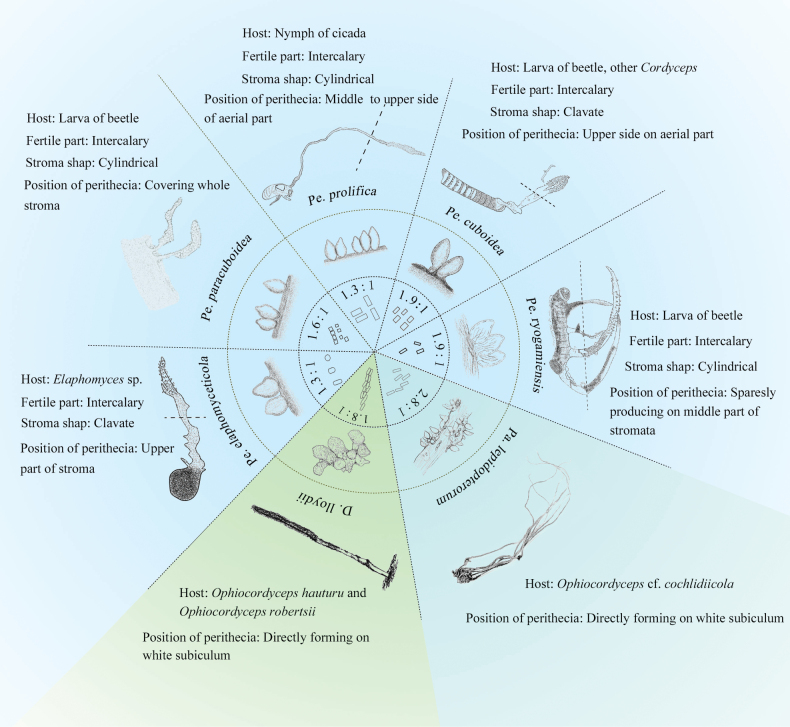
Morphological comparison of *Paradingleyomyces*, *Dingleyomyces* and *Perennicordyceps*. In *Perennicordyceps*, the dotted line below indicates burial in soil or woods. *Pe.cuboidea*, *Pe.prolifica*, *Pe.paracuboidea* and *Pe.ryogamiensis* were redrawn from Ban et al. (2009) and *Pe.elaphomyceticola* was redrawn from Xiao et al. (2023). *Pa.lepidopterorum* is a newly described species in this study. *D.lloydii* was redrawn from Johnston and Park (2023).

*Perennicordyceps* currently comprises six species, four identified based on their sexual morphology and two based on their asexual morphology. We have compared the sexual characteristics of *Pa.lepidopterorum* with the four sexual species of *Perennicordyceps*, as depicted in Fig. 7. Several distinctions between *Paradingleyomyces* and *Perennicordyceps* are observed: 1) *Pa.lepidopterorum* is characterized by the absence of stromata, while *Perennicordyceps* species exhibit branched, cylindrical to clavate, rhizomorphic stromata; 2) The host of *Pa.lepidopterorum* is Ophiocordycepscf.cochlidiicola, whereas *Perennicordyceps* parasitize a broader range of host, including insect and fungi; 3) The perithecia of *Pa.lepidopterorum* form on a white subiculum and are distributed over the entire stromata of the host fungus, while in *Perennicordyceps* species, perithecia are densely formed from the middle to the upper part of the stromata; 4) The length-to-width ratio of secondary ascospores in *Pa.lepidopterorum* is 2.8: 1, which is greater than that observed in *Perennicordyceps* species. Consequently, we introduced *Paradingleyomyces* as a distinct genus rather than categorizing it within *Perennicordyceps*. Although *Dingleyomyces* has shares the poorly developed stromata connecting crowed perithecia to the stromata of *Ophiocordycepshauturu* and *Ophiocordycepsrobertsii*, giving a similar appearance to *Pa.lepidopterorum*, multigene phylogeny revealed a paraphyletic relationship between *Dingleyomyces* and *Paradingleyomyces*. Therefore, the establishment of *Paradingleyomyces* is well-supported by both morphological observations and phylogenetic analysis. The asexual morph of *Paradingleyomyces* is currently unknown, and future efforts should focus on exploring more hidden species within this genus.

*Polycephalomyces* was initially classified under Hypocreales*incertae sedis* (Kepler et al. 2013; Matočec et al. 2014). Up to 25 species were assigned to *Polycephalomyces*, but later some were transferred to *Pleurocordyceps* and *Perennicordyceps*, remaining eight species: *Po.albiramus*, *Po.formosus*, *Po.ramosus*, *Po.tomentosus*, *Po.baltica*, *Po.cylindrosporus*, *Po.ditmarii*, and *Po.paludosus* (Xiao et al. 2023). However, the phylogenetic relationships of the latter four species remain unclear due to a lack of molecular data. Notably, *Po.ramosus* and *Po.tomentosus* group with species of *Pleurocordyceps* in the study of Xiao et al. (2023) and this phylogenetic relationship is also observed in our study. Thus, the taxonomic status of *Po.ramosus* and *Po.tomentosus* remains questionable. In this study, we introduce a new species, *Polycephalomycestengchongensis*, which is parasitic on Perennicordycepscf.elaphomyceticola from Tengchong County, Yunnan Province, China. This new species is distinguished by a unique combination of features, including its host association, synnemata lacking a stipe and fertile head, and the presence of dimorphic phialides and conidia (see Table 3). The finding of *Po.tengchongensis* adds to the morphological diversity within the genus *Polycephalomyces*.

**Table 3.** Distinguishing characteristics between *Po.tengchongensis* and other *Polycephalomyces* species.

Species	Host	Synnemata	Phialides (µm)	Conidia (µm)	Reference
***Po.tengchongensis***	**Perennicordycepscf.elaphomyceticola (Hypocreales, Polycephalomycetaceae)**	**Non-stipitate, without fertile head**	**Two-type, α-phialides 9–20 × 1–2, subulate; β-phialides 18–44 × 1–3, lanceolate**	**Two-type, α-conidia 1–3, globose; β-conidia 3–7 × 1.5–3, ellipsoidal to broadly fusiform**	**This study**
*Po.albiramus*	*Gryllotalpa* sp. (Orthoptera)	Stipitate, without fertile head	One-type, 12.8–18.3 × 1–2.2, narrowly subulate, awl-shaped	One-type 2.1–3.2 × 0.9–1.2, cylindrical to obovoid or subglobose	Xiao et al. (2023)
*Po.baltica*	Nymph or short-winged female bark louse	Stipitate, with fertile head	One-type, 3–4 long, flask-shaped	One-type, 3–4, globose	Poinar and Vega (2020)
*Po.cylindrosporus*	Coleoptera, Formicidae and Pentatomidae	Stipitate, with fertile head	One-type, 7–25 long	One-type, 2.5–4, cylindrical to bacilliform	Matočec et al. (2014)
*Po.ditmarii*	*Paravespulavulgaris* (Wasp), fly	Stipitate, with fertile head	One-type, 20–37 × 1.5–2.5, elongate, cylindrical, attenuating at the apex	One-type, 2.2–3 × 1.3–1.6, globose to subglobose to clavate	Van Vooren and Audibert (2005), Xiao et al. (2023)
*Po.formosus*	Coleopteran larvae or *Ophiocordycepsbarnesii*	Stipitate, with fertile head	One-type, 6–25 × 1–1.2, cylindrical, tapering gradually	One-type, 2.5–3.2 × 1–1.2, ellipsoidal or ovoid	Kobayasi (1941)
*Po.ramosus*	Lepidopteran larvae or *Hirsutellaguignardii*	Stipitate, with fertile head	Two-type, α-phialides 7–24 long, 1–2 at basal wide, cylindrical to narrowly lageniform; β-phialides 6–27 long, 2–3.5 at basal wide, 0.5–1 at neck width, narrowly lageniform or subulate	Two-type, α-conidia 2.4–3.2 × 1.6–2.4, ovoid; β-conidia 3.2–4 × 1.6–2, fusiform	Seifert (1985), Bischof et al. (2003)
*Po.paludosus*	Lepidopteran larva	Stipitate, with fertile head	One-type, 2–20 long, 1–1.5 at basal wide, subulate	One-type, 8–2.5 × 1.1–1.3, obovoid	Mains (1948)
*Po.tomentosus*	Myxomycetes	–	–	Three-type, globose or ellipsoidal or cylindrical	Seifert (1985)

*Pleurocordyceps* was introduced by Wang et al. (2021) to accommodate ten species: *Pleurocordycepsagarica*, *Pl.aurantiacus*, *Pl.lianzhouensis*, *Pl.marginaliradians*, *Pl.nipponica*, *Pl.onorei*, *Pl.phaothaiensis*, *Pl.ramosopulvinatus*, *Pl.sinensis*, and *Pl.yunnanensis*, based on phylogenetic analysis. Wei et al. (2022) added a new species *Pleurocordycepsophiocordycipiticola* which parasitizes *Ophiocordycepscylindrospora* in Thailand. Xiao et al. (2023) introduced five additional species to this genus, including *Pl.heilongtanensis*, *Pl.lanceolata*, *Pl.nutantis*, *Pl.parvicapitata* and *Pl.vitellina*. Currently, *Pleurocordyceps* comprises 16 species, all of which have been verified by molecular phylogeny. The sexual morph of *Pleurocordyceps* is characterized by stipitate, fibrous stromata that produce pale yellow to yellow fertile cushion either laterally or terminally, with immersed ostiolate perithecia, cylindrical asci, filiform disarticulating ascospores and cylindrical secondary ascospores. The asexual morph is characterized by stipitate, non-stipitate, or pulvinate synnemata, with or without fertile heads, generally displaying dimorphic phialides and conidia. Sexual morphs have been identified in six species including *Pl.marginaliradians* (Xiao et al. 2018), *Pl.nipponica*, *Pl.onorei* (Crous et al. 2017a), *Pl.parvicapitata* (Xiao et al. 2023), *Pl.phaothaiensis* (Crous et al. 2017b) and *Pl.ramosopulvinata* (Wang et al. 2021). The remaining 10 species of *Pleurocordyceps* have been described based on their asexual morphs. In this study, we report the sexual morph of *Pl.yunnanensis* from *Ophiocordycepsnutans* for the first time, collected from the same location as the type specimen. Ecologically, *Pleurocordyceps* species are particularly prone to infecting *Ophiocordyceps* or *Perennicordyceps* species, as well as their insect or fungal hosts. For instance, *Pl.parvicapitata* is known to infect *Perennicordycepselaphomyceticola* and its host *Elaphomyces* sp. at the same region (Xiao et al. 2023). In this study, we once again obtained *Pl.parvicapitata* which infects both *Pe.elaphomyceticola* and its host *Elaphomyces* sp. from Tengchong County, Yunnan Province. This finding indicates that *Pl.parvicapitata* may be specific to *Pe.elaphomyceticola* and *Elaphomyces* sp. Additionally, we are the first to isolate and observe dimorphic phialides and conidia in *Pl.parvicapitata*, while Xiao et al. (2023) reported only one type of phialides and conidia from dried specimen. Therefore, the presence of dimorphs phialides and conidia should not be considered a reliable feature for species demarcation within *Pleurocordyceps*.

## Supplementary Material

XML Treatment for Paradingleyomyces

XML Treatment for Paradingleyomyces lepidopterorum

XML Treatment for Polycephalomyces tengchongensis

XML Treatment for Pleurocordyceps parvicapitata

XML Treatment for Pleurocordyceps yunnanensis

## ﻿Additional information

### Author ORCIDs

Yi Wang https://orcid.org/0009-0006-5412-7893

De-Ping Wei https://orcid.org/0000-0002-3740-0142

Xing-Can Peng https://orcid.org/0000-0002-7271-7639

Ji-Chuan Kang https://orcid.org/0000-0002-6294-5793

Xian Zhang https://orcid.org/0009-0008-0919-4303

Putarak Chomnunti https://orcid.org/0000-0003-2989-1735

Ting-Chi Wen https://orcid.org/0000-0003-1744-5869

### Data availability

All of the data that support the findings of this study are available in the main text.
